# Case report: Analysis of novel compound heterozygous *TPP1* variants in a Chinese patient with neuronal ceroid lipofuscinosis type 2

**DOI:** 10.3389/fgene.2022.937485

**Published:** 2022-08-31

**Authors:** Sui-Bing Miao, Hui Guo, De-Xian Kong, Yuan-Yuan Zhao, Shu-Hong Pan, Yan Jiang, Xing Gao, Xiao-Hua Wu

**Affiliations:** ^1^ Key Laboratory of Maternal and Fetal Medicine of Hebei Province, Institute of Reproductive Medicine of Shijiazhuang, The Fourth Hospital of Shijiazhuang Affiliated to Hebei Medical University, Shijiazhuang, China; ^2^ Department of Obstetrics and Gynecology, The Fourth Affiliated Hospital of Hebei Medical University Shijiazhuang, Shijiazhuang, China; ^3^ Department of Endocrinology, The Fourth Affiliated Hospital of Hebei Medical University Shijiazhuang, Shijiazhuang, China; ^4^ Center of Reproductive Medicine, The Fourth Hospital of Shijiazhuang Affiliated to Hebei Medical University, Shijiazhuang, China

**Keywords:** neuronal ceroid lipofuscinosis type 2 (CLN2), tripeptidyl peptidase I (TPP1), variant, molecular analysis, splicing assay

## Abstract

Neuronal ceroid lipofuscinosis type 2 (CLN2) is an autosomal recessive neurodegenerative disease caused by variants in the *TPP1* gene that lead to the deficiency of the lysosomal enzyme tripeptidyl peptidase I (TPP1) activity. Herein, we report a rare case of CLN2 caused by two novel variants of *TPP1*. The patient presented with seizures at onset, followed by progressive cognitive impairment, motor decline, and vision loss. Novel compound heterozygous variants, c.544_545del and c.230-3C>G, in *TPP1* were identified by whole-exome sequencing. The variant assessment showed that the c.544_545del is a frameshift variant mediating mRNA decay and that c.230-3C>G is a splice variant generating aberrantly spliced TPP1 mRNA, as confirmed by a Splicing Reporter Minigene assay. In conclusion, clinical history, variant assessment, and molecular analyses demonstrate that the novel compound heterozygous variants are responsible for CLN2 disease in this patient. This study expands the mutation spectrum of *TPP1*.

## Introduction

Neuronal ceroid lipofuscinoses (NCL; CLN) are a clinically and genetically heterogeneous group of autosomal recessive progressive neurodegenerative disorders characterized by the accumulation of autofluorescent lipopigment storage material in lysosomes. They have an estimated worldwide incidence of 1–4/100,000 (Simonati et al., 2017). The main clinical features of this disease include epileptic seizures, cognitive decline, motor deterioration, progressive visual failure, and shortened life expectancy (Johnson et al., 2019). NCLs are classified into four major types: infantile, late-infantile, juvenile, and adult-onset, by age at onset. Thirteen causal genes have been identified so far (Gardner and Mole, 2021).

Neuronal ceroid lipofuscinosis type 2 (CLN2), also known as classical late-infantile neuronal ceroid lipofuscinosis (LINCL), is caused by mutations in *TPP1*, which encodes lysosomal tripeptidyl peptidase I (TPP1) (Yu et al., 2015). *TPP1* is mapped to chromosome 11p15. It consists of 13 exons and has a length of 13,696 bp (NG_008653.1). To date, 155 unique variants in *TPP1* have been identified (www.ucl.ac.uk/ncl-disease/mutation-and-patient-database). TPP1 is a lysosomal enzyme of 563 amino acids that specifically removes three amino acids from the N termini of proteins undergoing degradation in lysosomes (Vines and Warburton, 1999; Lin et al., 2001). Deficiency of TPP1 results in the accumulation of autofluorescent lipopigments in lysosomes, which is the typical pathomorphological change of all NCLs (McLaren et al., 2019).

Herein, we report a case of CLN2 with two novel *TPP1* variants, one frameshift and one splice variant, from a Chinese family. The variants were classified as pathogenic and likely pathogenic by analyzing their functional consequences and according to the American College of Medical Genetics and Genomics (ACMG) guidelines. Our study expands the mutation spectrum for *TPP1* and provides the functional consequences of the novel splice variant.

## Methods

### Whole-exome sequencing

Genomic DNA was extracted from peripheral blood by the Blood DNA Kit V2 (CW2553S, Cowin Bio, China). DNA samples were quantified with a Qubit dsDNA HS Assay Kit (Q32851, Invitrogen, United States). The quantified DNA samples were fragmented into 200–300 bp fragments and then processed for DNA library preparation with a KAPA Library Preparation Kit (KR0453, Roche, United States) according to the manufacturer’s protocol. Hybridization of the prepared libraries to capture probes was carried out according to the instructions for the xGen Exome Research Panel v2.0 (Integrated DNA Technologies Inc., United States). The captured DNA samples were sequenced on a HiSeq 2500 platform (Illumina, United States).

### Bioinformatics analysis

Raw data were processed by Fastp to remove adapters and filter low-quality reads. The qualified reads were aligned to the human reference sequence GRCh37/hg19 by BWA. Single nucleotide variants and insertions and deletions were called by GATK. Population and disease databases were used to obtain the frequencies of variants in large populations. The variants with allele frequency > 1% were filtered out. The ClinVar and HGMD databases were queried to identify previously reported cases with the variants. Multiple *in silico* algorithms were utilized to predict the pathogenicity of the candidate variants. Candidate variants were classified according to the ACMG guidelines (Richards et al., 2015). The version of RefSeq transcript accession used in the *TPP1* variant assessment was NM_000391.4.

### Sanger sequencing

Sanger sequencing was performed to validate the variants identified by WES. PCR primers were designed by Primer Premier 5.0 and are available upon request. PCR amplification was conducted with standard methods. The PCR products were purified and sequenced with an ABI 3730 DNA Sequencer (Applied Biosystems, United States), and the sequencing results were analyzed with Chromas Lite v2.01.

### Splicing Reporter Minigene assay

Genomic segments encompassing position c.230-3 (NM_000391.4) along with flanking intronic sequences spanning exons 3–5 and introns 3–4 in *TPP1* were amplified by PCR from the genomic DNA of the patient’s mother, who was the c.230-3C>G variant carrier. The wild-type (WT) c.230-3C and mutant (MT) c.230-3G sequences were then cloned into the minigene vector pMini-CopGFP (Hitrobio.tech, China) with a ClonExpress II One Step Cloning Kit (Vazyme, Nanjing, China). The following primers were used to construct the vectors: *TPP1*-F, AAG​CTT​GGT​ACC​GAG​CTC​GGA​TCC​GCT​GCC​CCC​AGG​CTG​GGT​GTC​CCT​GG; *TPP1*-R, TTA​AAC​GGG​CCC​TCT​AGA​CTC​GAG​CAA​AGT​CCA​CAT​GGG​GGG​CCA​AGG​CC. WT and MT constructs were identified by sequencing. The WT and MT plasmids were transfected into the HEK293T cell line for 48 h. Total RNA was extracted using TRIzol reagent (Cowin Biotech Co., Ltd., Taizhou, China). cDNA synthesis was performed using a HiScript II 1st Strand cDNA Synthesis Kit (R212-01, Vazyme Biotech, China). PCR amplifications were performed with the primers F-GGCTAACTAGAGAACCCACTGCTTA and R- CAA​AGT​CCA​CAT​GGG​GGG​CCA​A. The PCR products were separated by electrophoresis and analyzed by Sanger sequencing (Gaildrat et al., 2010).

## Results

### Case presentation

**FIGURE 1:**
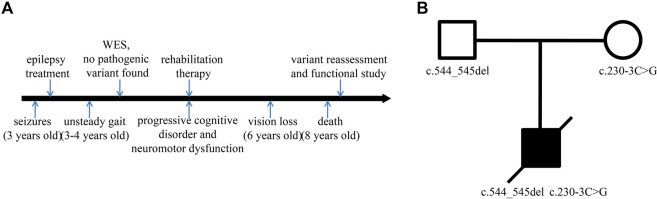
Timeline and family pedigree. **(A)** Timeline of disease and diagnosis course. **(B)** The parents were healthy with an unremarkable family history, but the proband presented with symptoms of CLN2.

The patient, a male with unremarkable antenatal and perinatal history, was taken to the hospital for the treatment of seizures at the age of 3. Until then, he had developed normally. Subsequently, progressive cognitive disorder, neuromotor dysfunction, and vision problems appeared gradually. Unsteady gait was observed at the age of 3–4 years, followed by increasing difficulties in walking, talking, and swallowing. His conditions continued to deteriorate over time. His sight and ability to stand and vocalize were lost at a later stage, and he ultimately died at the age of 8 (Figure 1A). CT showed mild brain atrophy, and MRI revealed that the T2 signal of the bilateral posterior horn of the lateral ventricle was slightly extended. EEG demonstrated a slow and irregular rhythm of background and epileptic discharges. The patient had been treated with carbamazepine and received rehabilitation therapy, but the treatments were ineffective. The pedigree of the family is shown in Figure 1B. The proband was born to healthy parents with an unremarkable family history.

### Variant analysis

**FIGURE 2:**
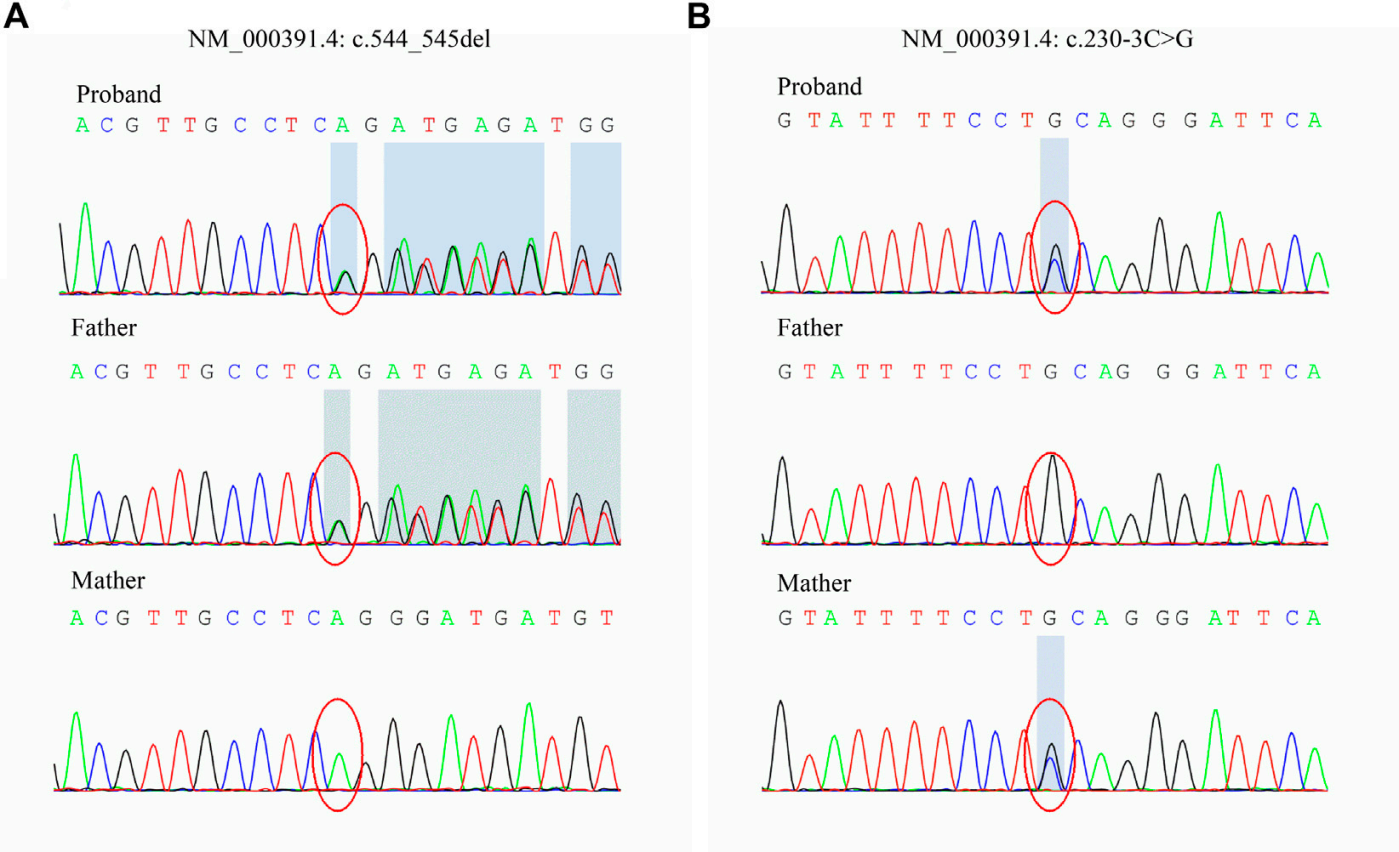
Sanger sequencing of the *TPP1* gene in the family. **(A)** The c.544_545del variant is carried by the proband and father. **(B)** The c.230-3C>G variant is carried by the proband and mother.

To explore the underlying genetic basis, WES was carried out to examine the exome of the patient. Two novel variants, c.544_545del and c.230-3C>G, in *TPP1* were found. The variants were then confirmed in the family by Sanger sequencing. The sequencing results showed that the c.544_545del variant was inherited from the father and that the c.230-3C>G variant was inherited from the mother (Figure 2). Next, the variants were classified according to ACMG guidelines (Richards et al., 2015). The c.544_545del variant was interpreted as pathogenic. The available evidence supporting this interpretation is as follows: it is a frameshift variant and nonsense-mediated mRNA decay (NMD) is predicted to occur (PVS1). The frequency of this variant is extremely low in the general population (PM2_Supporting). The phenotype of the patient is specific to the disease related to the gene (PP4). However, the pathogenicity of the other variant, c.230-3C>G, was predicted to be of uncertain significance based on available evidence. The variant was detected in trans with a pathogenic variant (PM3) and predicted to be deleterious by multiple lines of computational evidence (PP3) and PM2_Supporting and PP4.

**FIGURE 3:**
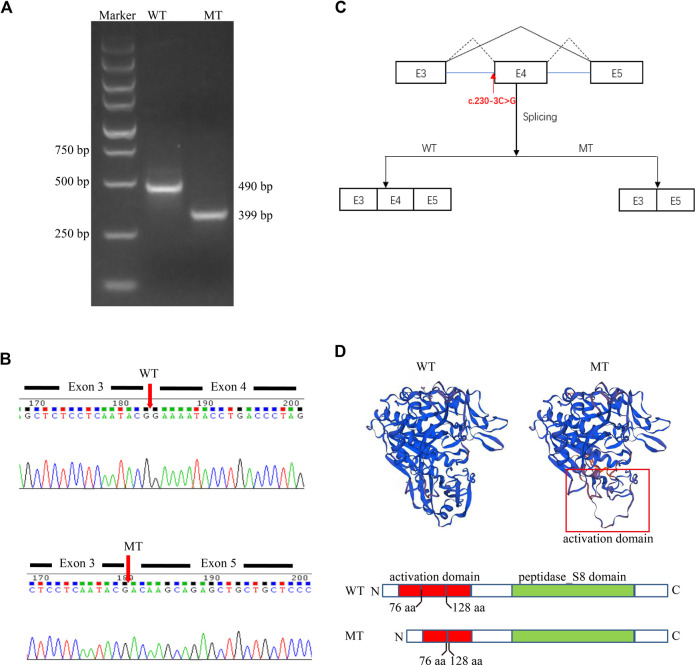
A Splicing Reporter Minigene assay was performed to determine the impact of the variant c.230-3C>G variant on transcript splicing. **(A)** Electrophoresis of PCR-amplified target fragments in WT and MT constructs. **(B)** Sanger sequencing of gel-excised bands. **(C)** Schematic diagram showing the complete skipping of the exon 4 due to the aberrant splicing induced by the variant c.230-3C>G variant. **(D)** Protein diagram and protein modeling with SWISS-MODEL (https://swissmodel.expasy.org/).

Given that the proband is presented with typical symptoms of CLN2, which follows an autosomal recessive inheritance pattern (Goebel and Sharp, 1998), we speculated that the variant c.230-3C>G might also be pathogenic. To further clarify the pathological significance of the c.230-3C>G variant, we determined the impact of the variant on transcript splicing using the Splicing Reporter Minigene assay (Hartikainen et al., 1999; Yuan et al., 2021). The RT-PCR products were analyzed by electrophoresis, and the results showed that the length of the PCR product was shortened in the MT construct (Figure 3A), indicating that aberrant splicing occurred. The sequencing result of the excised gel bands confirmed the absence of the sequence of exon 4 of 151 nt in length in the MT construct (Figure 3B). The Minigene assay demonstrated that the c.230-3C>G substitution abrogates the intron 3 canonical acceptor splice site and results in the skipping of exon 4 (c.230_380del) (Figure 3C). This alteration is predicted to lead to an in-frame deletion of amino acids and changes in protein structure (Figure 3D). Thus, we could add PS3 evidence (well-established *in vitro* or *in vivo* functional studies supportive of a damaging effect on the gene or gene product) for the variant interpretation, and the c.230-3C>G variant could be classified as likely pathogenic accordingly.

## Discussion

CLN2 is currently believed to be caused by the loss or reduction of TPP1 activity due to mutations in *TPP1* (Wang et al., 2011). This report presents a patient with classical LINCL manifestations, including uncontrolled seizures, cognitive impairment, motor deterioration, and progressive vision loss. Two novel variants, c.544_545del and c.230-3C>G in *TPP1*, were identified by WES and Sanger sequencing. According to ACMG guidelines, the c.544_545del variant was classified as pathogenic based on available evidence, and c.230-3C>G was interpreted as likely pathogenic by the Splicing Reporter Minigene assay.

The structure of the TPP1 protein contains an activation domain (residues 33–176) and a catalytic domain (residues 257–496), which compose the proenzyme form of TPP1. Upon acidification, the inactive proenzyme form is transformed into a mature enzyme by releasing the catalytic domain from the C-shaped structure formed by the activation domain (Pal et al., 2009; Gardner et al., 2019). Although the pathological mechanisms underlying CLN2 disease remain unclear, the majority are likely linked to loss of enzyme activity (Gardner et al., 2019). The c.544_545del variant is predicted to cause protein truncation (p.L182Efs*5). This truncation leads to the loss of the whole catalytic domain or nonsense-mediated mRNA decay according to NMD mechanisms (Brogna and Wen, 2009; Kurosaki et al., 2019) (http://autopvs1.genetics.bgi.com/variant/hg19/11-6638347-CAG-C:NM_000391.4). The c.230-3C>G variant can result in the skipping of exon 4. Codons of residues 77–127 in the primary structure of the protein TPP1 are in exon 4. Hence, the deletion of exon 4 due to the splice variation can result in damage to the activation domain and abnormalities in activating the enzyme. Collectively, we speculate that the two novel variants could cause a loss of enzyme activity. This could lead to substrate degradation failure within lysosomes and cause lysosomal storage disease.

WES is now becoming the main method for detecting mutations; however, correct interpretation of the numerous detected mutations is still challenging (Suwinski et al., 2019). Compared with the sequencing process, bioinformatics analysis and variant interpretation are more critical in the diagnosis of rare diseases (Nguyen and Charlebois, 2015). Sequencing is just the beginning of a genetic diagnosis, which often requires multiple analysis steps. This process takes a long time, and clinicians usually need to maintain communication with the clinical laboratory during the diagnosis process. In this case, the variants of *TPP1* were overlooked at disease onset because the patient only showed the first clinical symptom of seizures when WES was conducted. With the typical symptoms of LINCL appearing, such as cognitive decline and motor deterioration, variant reassessment was performed, and the variants c.544_545del and c.230-3C>G were finally identified. However, because one of the variants was classified as having uncertain significance based on available evidence, we could not conclude the cause of the patient’s symptoms. Next, a functional study was conducted, confirming that the intron variant c.230-3C>G could cause exon loss due to splicing variation. Only then was the pathogenicity of both variants confirmed, and the diagnosis was made by a comprehensive analysis of the clinical features, history, and interpretation of the variants.

In summary, c.544_545del and c.230-3C>G were confirmed as the causative variants for CLN2. Our study expands the spectrum of *TPP1* variants and provides a classification of the pathogenicity of the variants. In addition, this report attempts to highlight the continuity and complexity of the diagnostic process of genetic diseases through this report.

## Data Availability

The datasets for this article are not publicly available due to concerns regarding participant/patient anonymity. Requests to access the datasets should be directed to the corresponding author.
